# Periprosthetic joint infection of the knee complicated by primary localized cutaneous amyloidosis: a case report

**DOI:** 10.3389/fmed.2025.1667350

**Published:** 2025-10-23

**Authors:** Xinxuan Lai, Qianjin Zhang, Fuxin Liu, Zhiqing Xu, Jinhua Guo, Hanglin Qiu, Fudong Xu, Zhikun Zhuang, Changyu Huang

**Affiliations:** Department of Orthopaedic Surgery, Quanzhou Orthopedic-traumatological Hospital, Quanzhou, China

**Keywords:** primary localized cutaneous amyloidosis, periprosthetic joint infection, total knee arthroplasty, treatment, antibiotic

## Abstract

**Background:**

Primary localized cutaneous amyloidosis (PLCA) is characterized by amyloid protein deposition in the skin, leading to pigmentation, papules, and itching. The etiology of PLCA remains unclear. Periprosthetic joint infection (PJI) post-total knee arthroplasty (TKA) poses challenges in management.

**Case presentation:**

A PLCA patient underwent TKA and developed acute PJI, necessitating multiple surgical interventions. Recurrence of infection symptoms prompted a two-stage revision surgery approach.

**Conclusion:**

Skin lesions in PLCA patients may potentially contribute to the risk PJI following arthroplasty. Strategies such as using tacrolimus for itching management, along with prophylactic antibiotics during PLCA flare-ups, could potentially aid in preventing and managing PJI in this patient population. Further research is needed to elucidate the relationship between PLCA and PJI post-arthroplasty.

## Introduction

Primary localized cutaneous amyloidosis (PLCA) is a rare chronic condition characterized by the deposition of amyloid protein in the skin (1). The deposition of amyloid protein can lead to skin pigmentation, papules, and nodules, often accompanied by severe itching (2). The exact etiology of PLCA remains unclear, potentially related to genetic factors, immune responses, and metabolic abnormalities (1). Periprosthetic joint infection (PJI) is a disastrous complication post-joint arthroplasty, often requiring multiple surgeries and prolonged antibiotic use, imposing significant burdens on patients (3). Here, we report a patient with PLCA undergoing total knee arthroplasty (TKA) who developed acute PJI post-TKA and underwent a failed debridement, antibiotics, and implant retention (DAIR), suggesting a potential connection between PLCA and PJI.

## Case report

**Figure 1:**
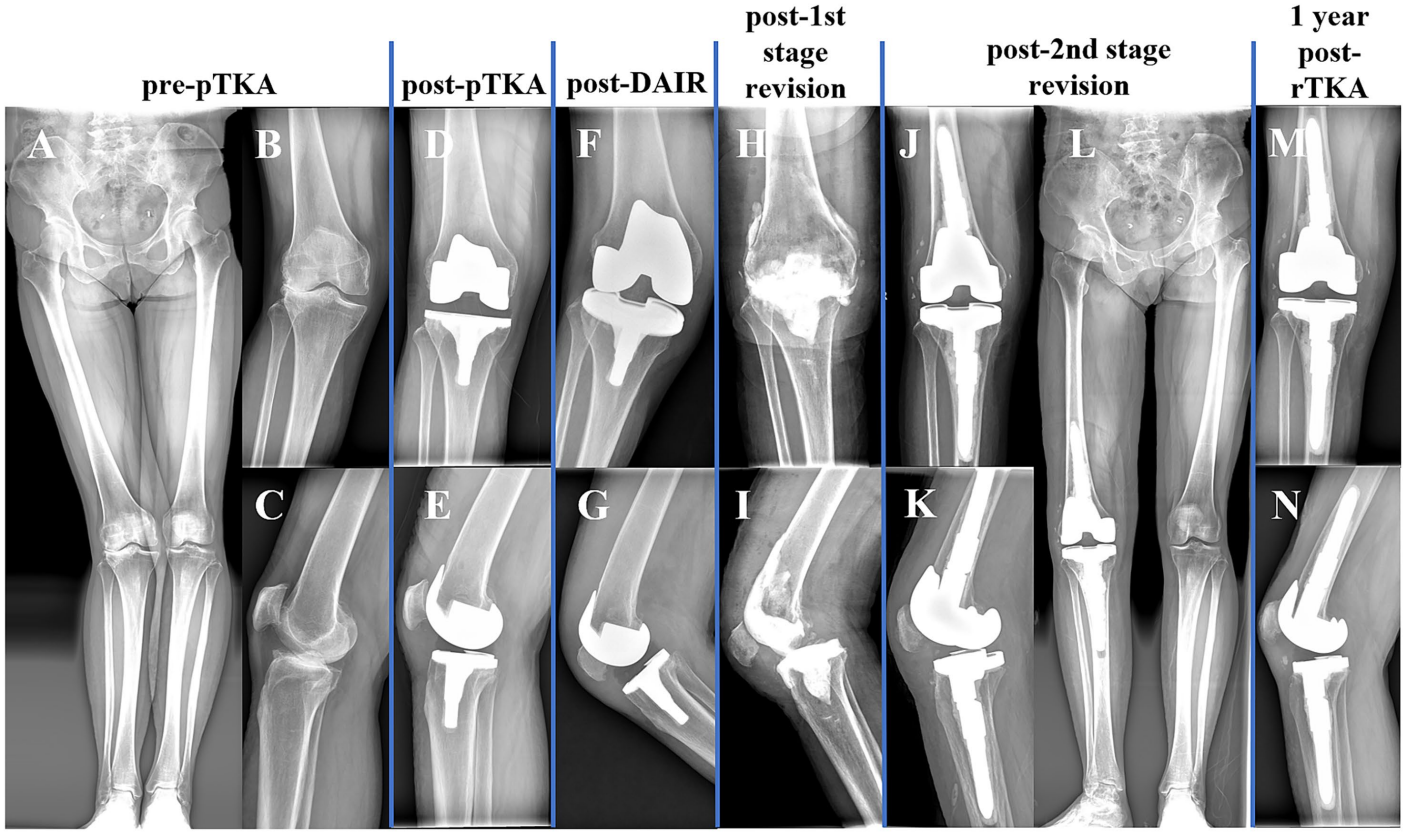
Patient knee joint X-ray images at different stages. **(A,B)** Before the primary total knee arthroplasty (pTKA); **(D,E)** Post pTKA; **(F,G)** Post-debridement, antibiotics, and implant retention (DAIR); **(H,I)** Post-1st stage revision; **(J,L)** Post-2nd stage revision; **(M,N)** One year post-TKA revision.

A 59-year-old female (BMI: 24.3 kg/m^2^) presented to our center due to recurrent right knee pain and limping, which had been ongoing for 5 years. X-rays revealed severe degeneration of her right knee (Figures 1A–C). Approximately 10 years ago, she developed pigment deposition and papules on both lower legs. A skin biopsy at a dermatology specialty hospital confirmed PLCA, for which she received treatment, albeit unsuccessfully. During active PLCA episodes, she experienced unbearable itching on both lower legs. According to her recollection, her deceased father had a similar condition on both lower legs. She was otherwise in good health.

**Figure 2:**
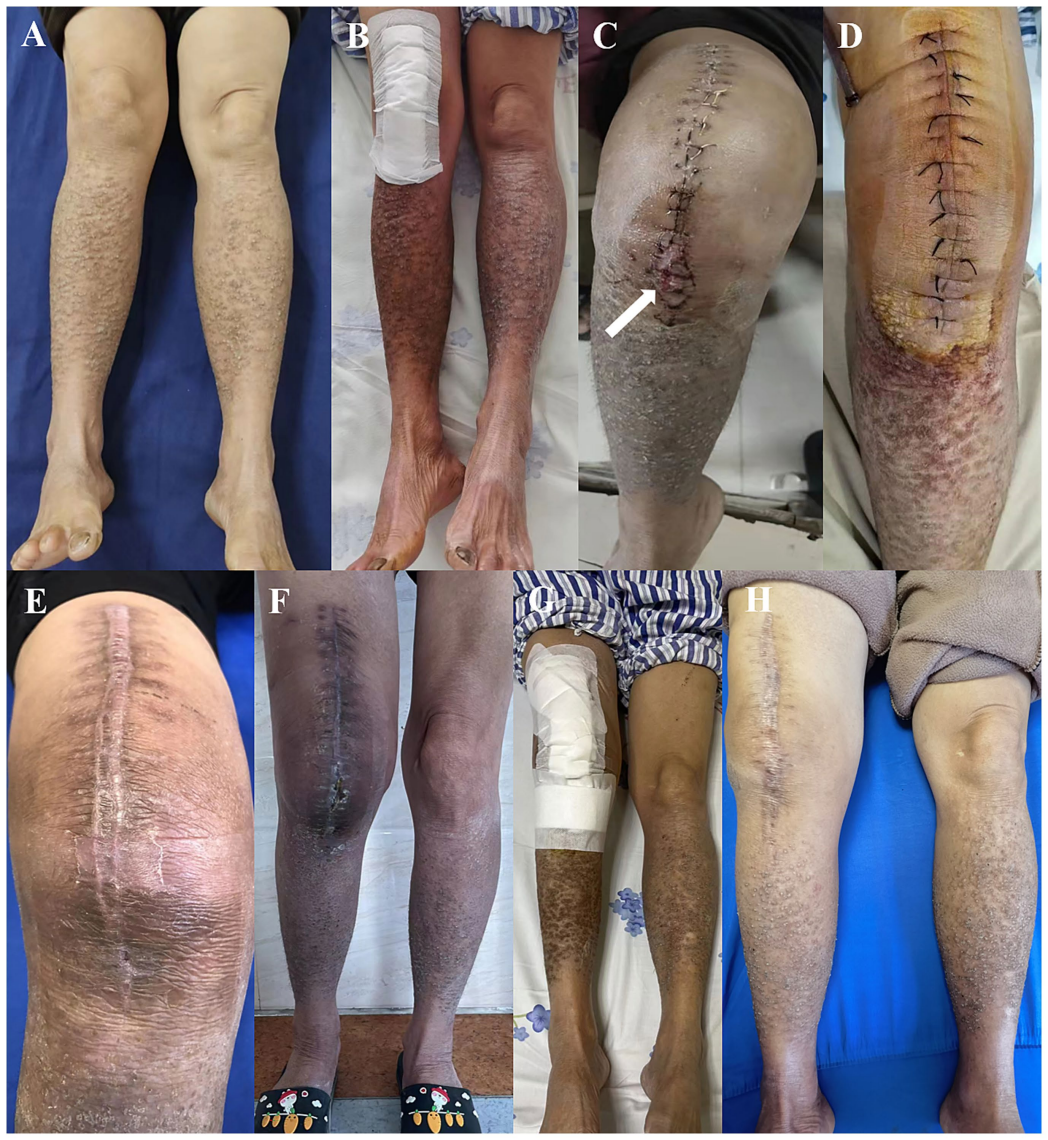
Patient skin presentations at various stages. **(A)** Before the primary total knee arthroplasty (pTKA); **(B)** Post pTKA; **(C)** Pre-debridement, antibiotics, and implant retention (DAIR); **(D)** Post-DAIR; **(E)** Pre-1st stage revision; **(F)** Post-1st stage revision; **(G)** Post-2nd stage revision surgery; **(H)** One year post-TKA revision.

Upon initial admission, her lower leg skin temperature was normal (Figure 2A). Subsequently, she underwent primary total knee arthroplasty (pTKA) on the right side (Figures 1D,E) and received prophylactic antibiotics (cefazolin) for 24 h.

On the third day post-pTKA, her lower leg skin temperature increased, accompanied by itching, leading to repeated scratching of both lower legs (Figure 2B). On the 12th day post-pTKA, her knee started to swell and become painful, with purulent discharge from the surgical incision observed on the 19th day post-surgery (Figure 2C). Joint fluid culture from arthrocentesis indicated *Staphylococcus aureus* infection. According to the 2018 ICM criteria (4), she was diagnosed with acute PJI of the knee and underwent DAIR with spacer exchange (Figures 1F,G, 2D). Subsequently, she received intravenous vancomycin (1 g, q12 hours) for 2 weeks followed by oral cefaclor (375 mg, BID) for 4 weeks. Due to the prevalence of COVID-19, she did not have regular follow-up visits.

Unfortunately, three months after the DAIR procedure, she experienced redness, swelling, and pain in her knee again. According to her recollection, she had recurrent itching on both lower legs following the DAIR, leading to repeated scratching, although she did not seek dermatological consultation. Four months post-DAIR, she returned to our center (Figure 2E), and purulent discharge was obtained from arthrocentesis of her knee, confirming *Staphylococcus aureus* infection. Consequently, she was diagnosed with chronic PJI, with the recurrent acute episodes of PLCA drawing the attention of the surgical team. After consulting a dermatologist, she started using 0.1% tacrolimus cream locally. Subsequently, a two-stage revision surgery plan was devised for her. During the 1st revision, she used an antibiotic (vancomycin) cement spacer (Figures 1H,I, 2F) and received 2 weeks of intravenous vancomycin and 6 weeks of oral cefaclor. Additionally, she was advised to resume oral cefaclor if experiencing itching and scratching during antibiotic-free intervals. Six months post-1st revision, she underwent a 2nd revision (Figures 1J–L, 2G) and received a similar antibiotic regimen. Similarly, she was recommended to restart cefaclor if experiencing recurrent itching on both lower legs after discontinuing antibiotics. One year post-2nd stage surgery, she had regained normal functional ability, with slight limitation in knee extension, persistent PLCA on both lower limbs, but her knee function was acceptable (with a Knee Society Score of 80), showing no signs of infection recurrence (Figures 1M,N, 2H).

## Discussion

PJI is one of the most serious complications following TKA, with an incidence rate of approximately 2% (5). Even after undergoing multiple surgical interventions and prolonged antibiotic therapy, the treatment success rate of PJI remains between 71.6 to 80.6% (6). The microbial pathways leading to PJI are commonly believed to be through either seeding at the surgical site or hematogenous dissemination. Research by Bengtson et al. suggests that skin damage is the primary source of hematogenous infection post-TKA (7). In this case, we report a patient with knee osteoarthritis complicating with PLCA. The repetitive scratching induced by PLCA led to multiple skin lesions on the lower legs. We hypothesize that pathogenic bacteria invaded through these lesions and disseminated via the bloodstream to the prosthetic joint, causing the occurrence of PJI. Although we did not conduct homogeneity testing for *Staphylococcus aureus*, we speculate that these recurrent skin lesions may have contributed to the failure of DAIR. To our knowledge, this may be the first reported case of PJI associated with PLCA.

Beyond PLCA, other chronic dermatological conditions characterized by pruritus and skin breakdown may similarly predispose patients to PJI. Conditions like psoriasis and chronic eczema involve a compromised skin barrier and repetitive scratching, which could theoretically facilitate bacterial entry and subsequent hematogenous seeding to prosthetic joints (8). While direct epidemiological studies linking these conditions to PJI are limited, the shared mechanism of impaired skin integrity supports the need for heightened vigilance and similar prophylactic strategies in patients with chronic dermatoses undergoing arthroplasty.

PLCA often occurs around the age of 50 and is more common in populations from Southeast Asia and South America. PLCA primarily affects specific areas of the skin and does not involve systemic amyloidosis. The typical presentation of PLCA includes multiple discrete hyperkeratotic papules, most commonly seen on the shins, and can cause intense itching (9). In this case, acute flare-ups of PLCA were characterized by intense pruritus, a noticeable increase in local skin temperature, erythema, and the appearance of new papules or excoriations due to scratching. Genetic factors may play a role in the pathogenesis of PLCA (1), as indicated by the patient’s description of a similar condition in her father. However, the exact cause of her PLCA remains unclear.

The common treatments for PLCA include local application of corticosteroids, antihistamines, local immunomodulators such as tacrolimus, and narrowband ultraviolet B phototherapy. However, none of these treatments seem to provide a cure. Therefore, the primary goal of treating PLCA currently focuses on symptom relief, particularly alleviating itching and breaking the itch-scratch cycle (10). In this case, due to the presence of infection, corticosteroids were not used, and tacrolimus was employed, significantly reducing the patient’s itching. This indicates that tacrolimus may be effective in patients with PLCA complicating with PJI. Additionally, to prevent transient bacteremia due to recurrent skin lesions, we recommend prophylactic oral antibiotics for patients during PLCA itch episodes. In this case, oral cefaclor was used empirically during PLCA flare-ups due to its activity against *Staphylococcus aureus*, the most common pathogen in PJI. However, the selection of prophylactic antibiotics should ideally be guided by prior microbiological results and local antibiograms. Within one year post-rTKA, despite recurrent PLCA episodes, the patient did not experience a recurrence of PJI.

In conclusion, skin lesions in patients with PLCA may contribute to the occurrence of PJI following TKA. For PLCA patients undergoing TKA, efforts should be made to avoid or alleviate scratching-induced by itching during the perioperative period, with the local application of tacrolimus potentially being effective. During PLCA flare-ups, prophylactic oral antibiotics may help prevent the occurrence and recurrence of PJI.

## Data Availability

The datasets used and analyzed during the current study are available from the corresponding author on reasonable request.
